# Knockdown of interleukin‐6 plays a neuroprotective role against hypoxia‐ischemia in neonatal rats via inhibition of caspase 3 and Bcl‐2‐associated X protein signaling pathway

**DOI:** 10.1002/ibra.12067

**Published:** 2022-09-18

**Authors:** Xiu Yang, Ke‐Han Liao, Isaac B. Deng, Lan‐Chun Zhang

**Affiliations:** ^1^ Animal Model Research Center of Human Disease Kunming Medical University Kunming China; ^2^ School of Anesthesiology Southwest Medical University Luzhou China; ^3^ Health and Biomedical Innovation, Clinical and Health Sciences University of South Australia Adelaide South Australia Australia

**Keywords:** BAX, caspase 3, interleukin‐6, neonatal hypoxic‐ischemic encephalopathy

## Abstract

This study aimed to investigate the role of interleukin‐6 (IL‐6) in the pathogenesis of neonatal hypoxic‐ischemic encephalopathy (NHIE). Sprague‐Dawley (SD) rats were used for the establishment of hypoxic‐ischemic (HI) model. The Zea‐Longa scoring was used to evaluate the extent of the neurological deficits. Triphenyl tetrazolium chloride (TTC) staining was used to measure the volume of infarction in the brain following HI protocol. The expression of IL‐6 in the cortex and/or hippocampus at multiple time points after HI was examined by immunohistochemistry, western blotting and immunofluorescence. Moreover, small interfering RNAs (siRNA) were used to inhibit the expression of IL‐6 in‐vitro and in‐vivo, and the concomitant expression of the Bcl‐2 associated X protein (BAX) and caspase 3 was also measured. HI induced a significant brain damage, and these pathological changes were accompanied by IL‐6 upregulation which was found localized in cortical neurons. The inhibition of IL‐6 expression fostered neuronal and axonal growth, and a reduction in cellular apoptosis in cortical neuronal cultures, and cortex and hippocampus of neonatal rats. The expression of apoptotic markers such as BAX and caspase 3 was closely associated with IL‐6. Downregulation of IL‐6 could ameliorate HI‐induced deficiencies by mediating the expression of caspase 3 and BAX.

## Graphical abstract

Hypoxic‐ischemic (HI) induced significant brain damage, and these pathological changes were accompanied by IL‐6 upregulation. Decreased Interleukin (IL)‐6 improves the deficiencies in neurologic function and morphology induced by HI, and the potential mechanism may be closely related to the regulation of caspase 3 and Bcl‐2 associated X protein.

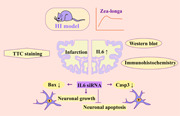

## 1 INTRODUCTION

Neonatal hypoxic‐ischemic encephalopathy (NHIE) is a brain dysfunction caused by neonate asphyxia,^1^ which can evolve into cerebral palsy, epilepsy, and cognitive disabilities,^2^, ^3^, ^4^ and it affects 1–5/1000 newborns worldwide. Hypoxia and ischemia (HI) interrupt the energy supply of cells which could result in cell injury.^5^ HI and reoxygenation/reperfusion produce a wide range of toxic chemicals such as free oxygen species and inflammatory cytokines, which could exacerbate cellular injury,.^6^ Studies have shown that rats exposed to longer hypoxic periods at post neonatal Day 3 suffered from encephalic and sensorimotor impairments that mimic those observed in preterm infants.^7^ Clinically, there lacks an effective treatment to halt or minimize HI‐induced brain dysfunction, emphasizing the need for new effective interventions.

Interleukin‐6 (IL‐6) is a proinflammatory cytokine^8^ associated with the mediation of inflammation. A multitude of studies have shown that inflammatory cytokines contribute to the pathogenesis of ischemia‐induced brain damage.^9^ Increased level of IL‐6 has been reported in several neurological diseases,^10^ suggesting the neuropathological role of IL‐6. Indeed, a study by Silveira and colleagues reported an increased level of IL‐6 in the cerebrospinal fluid and plasma of infants with NHIE. Another study unveiled that hypoxia‐induced IL‐6 production through activating mitogen‐activated protein kinase (MAPK), hypoxia‐inducible factor‐1 (HIF‐1), and nuclear factor‐kappa B (NF‐kappa B).^11^, ^12^ Cumulatively, all the aforementioned evidence suggests the involvement of IL‐6 in the pathogenesis of NHIE; however, the underlying molecular mechanisms are largely unknown. Some studies have shown that IL‐6 modulates the expression of genes involved in inflammation and apoptosis.^13^ Of note, it is well documented in a variety of studies that the levels of IL‐6 correlate with the expression of Bcl‐2 associated X protein (BAX) and caspase 3, which are apoptotic markers.^14^, ^15^, ^16^ During apoptosis, BAX undergoes a series of conformational changes simultaneously with mitochondrial changes, particularly the release of cytochrome C, leading to the activation of caspases which coordinate the efficient breakdown of dying cells.^17^ Currently, the detection of active caspase 3 in cells and tissues is the most recognized biochemical hallmark of apoptosis.^18^ The importance of IL‐6, Bax, and caspase 3 in NHIE is further illustrated by a study that showed that perillaldehyde ameliorated cerebral cortical ischemia‐reperfusion injury in rats by reducing the levels of IL‐6 along with BAX and caspase 3.^17^ This study is aimed at exploring the role of IL‐6 in NHIE‐induced neurological dysfunction and brain damage.

## 2 MATERIALS AND METHODS

### 2.1 Animal care

Timed pregnant female Sprague‐Dawley (SD) rats were bought from the Animal Centre of Kunming Medical University and placed in individual cages. Postnatal pups were put in their cage with food and water available at libitum in a 12‐h light/dark cycle. The SD rat pups (weighing 10–15 g) were used for this study at 7 days postnatal. All the experiments were carried out in accordance with the care and use of laboratory animals promulgated by the Ministry of Science and Technology of the People's Republic of China and approved by the Animal Care and Use Committee of Kunming Medical University (kmmu2019039). Operations on animals were in compliance with the National Institutes of Health Guide for the Care and Use of Laboratory Animals.

### 2.2 Establishment of HI model

The neonatal rats were deeply anesthetized with 3% isoflurane before any surgical procedure. Subsequently, a 0.5 cm skin incision was made in the midline of the neck, and the right common carotid artery (CCA) was exposed and permanently fused by Monopolar Microsurgery Electrocoagulator (Spring Medical Beauty Equipment co., LTD). The rats were returned to their mother to recover for 1h after surgery. After that, the pups were taken into a hypoxic chamber (8% O_2_, 92% N_2_) for 2 h to induce hypoxic injury, and then returned to their maternal rats. The neonatal rats in the sham group were anesthetized with 3% isoflurane before the CCA procedure without exposure to the hypoxic chamber.

### 2.3 Behavioral analysis

The Zea‐longa scoring was used to assess the neurological deficits of rats. The Zea‐longa scoring correlates with the severity of the neurological deficits. Briefly, the neurobehavioral scores of the rats were evaluated at 0, 4, 6, 8, 12, and 24 h after HI was induced.^19^ The scoring criteria were as follows: (1) 0 point (no neurological deficits—normal behavior); (2) one point (mild neurological deficits—the left forelimb can't be fully extended); (3) two points (moderate neurological deficits—rats cannot go straight and walk forward, and the body continues to turn to one side); (4) three points (severe neurological deficit—rats are unable to stand, felling to the left when standing); (5) four points (loss of consciousness).

### 2.4 Brain water content

The brains of rat pups were removed after deep anesthesia and separated into ipsilateral, contralateral, and cerebellum portions to measure the water content at 12 and 24 h after HI. Afterward, the wet weights of the separated brain segments were measured with electronic balance immediately upon collection. In contrast, the aforementioned brain segments were dried in an oven at 105°C for 24–36 h to reach a constant weight (the last two times weighing < 0.2 mg) then the dry weights were measured. Finally, the brain water content was calculated by the Elliot formula: the percentage of brain water content = (wet weight – dry weight)/wet weight × 100%.

### 2.5 Tissue collection

The brain tissues of the rat pups were collected at indicated time points after surgery. First, the rats were perfused with sterile saline followed by 4% paraformaldehyde (pH 7.4, 0.01 mol/L PBS). Subsequently, the brains were removed immediately after perfusion and placed in 4% paraformaldehyde fixative for more than 72 h. The brain samples for real‐time quantitative polymerase chain reaction (RT‐qPCR) and western blot (WB) were only perfused with sterile saline, and then stored at −80°C.

### 2.6 Triphenyl tetrazolium chloride (TTC) staining

TTC staining was performed to detect the cerebral infarct volume at 12 and 24 h after injury as previously described.^20^ The harvested brain tissues were frozen and then cut into five coronal slices at a thickness of 2 mm. Incubation with 2% TTC solution (Sigma, USA) was followed at 37°C for 5 min. The percentage of infarction was calculated with the following formula: corrected percentage of infarct volume = (contralateral hemispheric volume − ipsilateral noninfarcted volume)/contralateral hemispheric volume.

### 2.7 Hematoxylin and eosin (H&E) staining

The brain samples were first fixed in 4% paraformaldehyde solution before H&E staining. Subsequently, the frozen brains were sectioned into 50 μm thick sections using a freezing microtome (CM1860, Leica Microsystems). The prepared sections were stained with hematoxylin solution (Servicebio, G1005) for 1 min, and then washed under running water for 1 min. After that, the sections were immersed in eosin solution (Servicebio, G1005) for 2–3 min. Next, sections underwent dehydration and transparentization. The fields near the core infarcted area were captured for analysis under a light microscope (CX40, Shunyu).

### 2.8 Immunohistochemistry

An immunohistochemical assay was performed according to our previous publication.^21^ The tissue sections were incubated with IL‐6 primary antibody (rabbit anti‐IL‐6; 1:100; ZSGB‐BIO, China) for 18 h followed by corresponding secondary antibodies (goat anti‐rabbit; ZSGB‐BIO, China) for 2 h. The same protocol was adapted for the sham group with the exception that a PBS solution was used instead of the primary antibody. The optical density of IL‐6 in the tissues was measured by ImageJ pro software (NIH, the USA).

### 2.9 Primary culture of rat cortical neurons and PC12 cells

Primary cortical neurons were obtained from SD rats at 1‐day postnatal. Briefly, the cortices of the rats were harvested and sectioned into approximately 1 mm^3^ pieces. Subsequently, the tissue sections were digested with 0.25% trypsin (Gibco) at 37°C for 10 min and eluted with 10% fetal bovine serum (FBS, BSA, Gibco). The samples were centrifuged at 1500 rpm for 5 min, and the pellets were resuspended using a Hyclone medium containing Dulbecco's modified Eagle medium (DMEM)/HIGH GLUCOSE, 10% FBS, and 1% penicillin‐streptomycin solution). Next, the neurons were seeded at a density of 5 × 10^5^ cells/ml into 6‐well plates (Corning, USA) coated with poly‐d‐lysine and laminin (Sigma‐Aldrich), then incubated at 37°C with 5% CO_2_. The completed medium was replaced with 2% B27 (Invitrogen, CA) at 4 h after the incubation. The medium was changed the next day and then half change was made every 3 days. PC12 cells were obtained from American Type Culture Collection and cultured in DMEM (Cat. No. 10569‐010) supplemented with 10% FBS (Cat. No. 16000‐077). Cortical neurons were subjected to oxygen‐glucose deprivation (OGD) insult as previously described.^22^

### 2.10 Screening for the effective fragment of IL‐6 small interfering RNAs (siRNA) and transfection

To silence IL‐6 expression, three 3‐19 nucleotide sequences were designed to correspond to the IL‐6 reference sequence (NCBI, NM_031512.2). The three siRNAs that inhibit IL‐6 gene expression and one nonsense siRNA as negative control (NC) were designed and purchased from Ribobio Company. PC12 cells were assigned to the following groups: the normal group, reagent group, negative control group, and IL‐6 siRNA groups (F1, F2, and F3). The most efficient siRNA fragment was selected to be transfected into the cultured neurons. The nontargeting siRNA was transfected into neurons as an NC. Transfection was performed using SuperFectin II in vitro transfection reagent (Pufei Biotech). In addition, the morphologic changes of neurons after IL‐6 siRNA treatment were observed and documented by an inverted fluorescence Microscope.

### 2.11 Immunofluorescence staining of tissue section

Sections were washed three times (5 min each time) in PBS and incubated with 5% goat serum for 2 h at room temperature to block nonspecific binding. Subsequently, sections were incubated overnight at 4°C with the following primary antibodies: anti‐IL‐6 antibody (mouse, 1:100; Abcam), anti‐NeuN antibody (rabbit, 1:100; ZSGB‐BIO), anti‐GFAP antibody (rabbit, 1:100; ZSGB‐BIO) and anti‐caspase 3 antibody (rabbit, 1:100). Then, the sections were washed three times with PBS for 5 min each time, and incubated with the appropriate secondary antibodies: CY3 anti‐mouse IgG (red), Alexa Fluor 488 anti‐mouse IgG (green) and Alexa Fluor 488 anti‐rabbit IgG (green) for 2 h at 37°C. The sections were washed three times for 5 min each with PBS and stained with DAPI.

### 2.12 Immunofluorescence in vitro

The prepared cell slices were incubated with primary antibodies of Tuj1 (mouse; 1:100; Abclonal) overnight at 4°C, followed by incubation with secondary antibodies (DyLight 488, anti‐mouse IgG; 1:200; ZSGB‐BIO) for 1 h at 37°C. The images of five random fields were captured at 200 magnification under a fluorescent microscope. Quantification analysis was carried out using Image‐Pro Plus 6.0 software (MediaCybernetics).

### 2.13 Terminal‐deoxynucleoitidyl transferase mediated nick end labeling (TUNEL) staining

TUNEL reaction mixture of enzyme solution and labeling solution was added at a ratio of 1:9 (v/v), and the slices were stored at 4°C overnight in the dark. After washing with PBS, the neurons were stained with DAPI for 5 min at room temperature, and photographs were obtained using fluorescence microscopy (Leica, CM1860). The nuclei of apoptotic neurons were stained in red by TUNEL and all nuclei of neurons were stained in blue by DAPI. Apoptosis was quantified by determining the percentage of TUNEL/DAPI in the randomly selected fields using Image‐Pro Plus 6.0 software.

### 2.14 WB detection

WB was used to detect the protein expression of IL‐6 in the cortex and hippocampus. The cortical, and hippocampal tissues were lysed with RIPA Buffer (Beyotime), and then centrifuged at 12,000× g for 10 min at 4°C. We collected the supernatants and measured the total protein concentration using a microplate reader. The proteins were electrophoretically separated on a 10% sodium dodecyl sulfate‐polyacrylamide, and then were transferred to a polyvinylidene fluoride (PVDF) membrane. Afterward, the membrane was immersed in IL‐6 primary antibody solution (mouse, 1:200) for 24 h at 4°C, followed by a secondary antibody (goat anti‐mouse IgG, 1:5000) incubation for 2 h at 37°C. The Beta‐actin antibody was used as an internal control. The membrane was developed following the secondary antibody incubation and imaged using an ECL detection system (Amersham Pharmacia Biotech, Buckinghamshire). The densitometry analyses were performed using Image J software. The data is expressed as a ratio of the optical density (OD) values of the proteins of interest to the OD values of β‐actin.

### 2.15 RT‐qPCR detection

Total RNA was extracted from the cortex, hippocampus, and cultured neurons by Trizol reagent (Takara Bio Inc.), and then it was reversely transcribed into complementary DNA (cDNA) using the Revert Aid™ First Strand cDNA Synthesis kit (Invitrogen). The primer sequences for the genes detected are listed in Table 1. The reaction was performed in a thermal cycler (CFX96) in accordance with the following standard protocol: a cycle of 95°C for 3 min; 45 cycles of 95°C for 15 s (s) and annealing temperature at 60°C for 30 s. The threshold cycle of each sample was recorded, and data were analyzed by normalization to GAPDH values using the 2^−△△*Ct*^ method.

**Table 1:** Primer sequences of the detected genes

Genes	Sense	Antisense	Anealing (°C)
Interleukin (IL)‐6	AGAAGACCAGAGCAGATTTT	GAGAAAAGAGTTGTGCAATG	51
Bcl‐2 associated X protein (BAX)	TGGAGCTGCAGAGGATGATT	CAGGGCCTTGAGCACCAGTT	53
Caspase 3	CGGGTCATGGTTCATCCAGT	CTCAAATTCCGTGGCCACCT	52
GAPDH	GACATGCCGCCTGGAGAAAC	AGCCCAGGATGCCCTTTAGT	52

### 2.16 IL‐6 interference lentivirus injection in‐vivo

IL‐6 interference lentivirus (MSH028938‐HIVU6) was purchased from GeneCopoeia Company to construct an IL‐6 siRNA expression vector. The production of IL‐6 lentivirus and injection in neonatal rats was performed as described previously.^23^

### 2.17 Statistical analysis

All the data in the experiment were analyzed using SPSS 20.0, and are presented as mean ± SD. Data meeting the normal distribution were analyzed by one‐way analysis of variance (ANOVA) with Bonferroni analysis for data under equal variance conditions or Dunnett's analysis for data under uneven variance conditions. Data that don't meet normal distribution were analyzed by Kruskal–Wallis test. Statistical difference was deemed significant when *p* < 0.05.

## 3 RESULTS

### 3.1 HI induced cerebral damage and neurological dysfunctions

**Figure 1:**
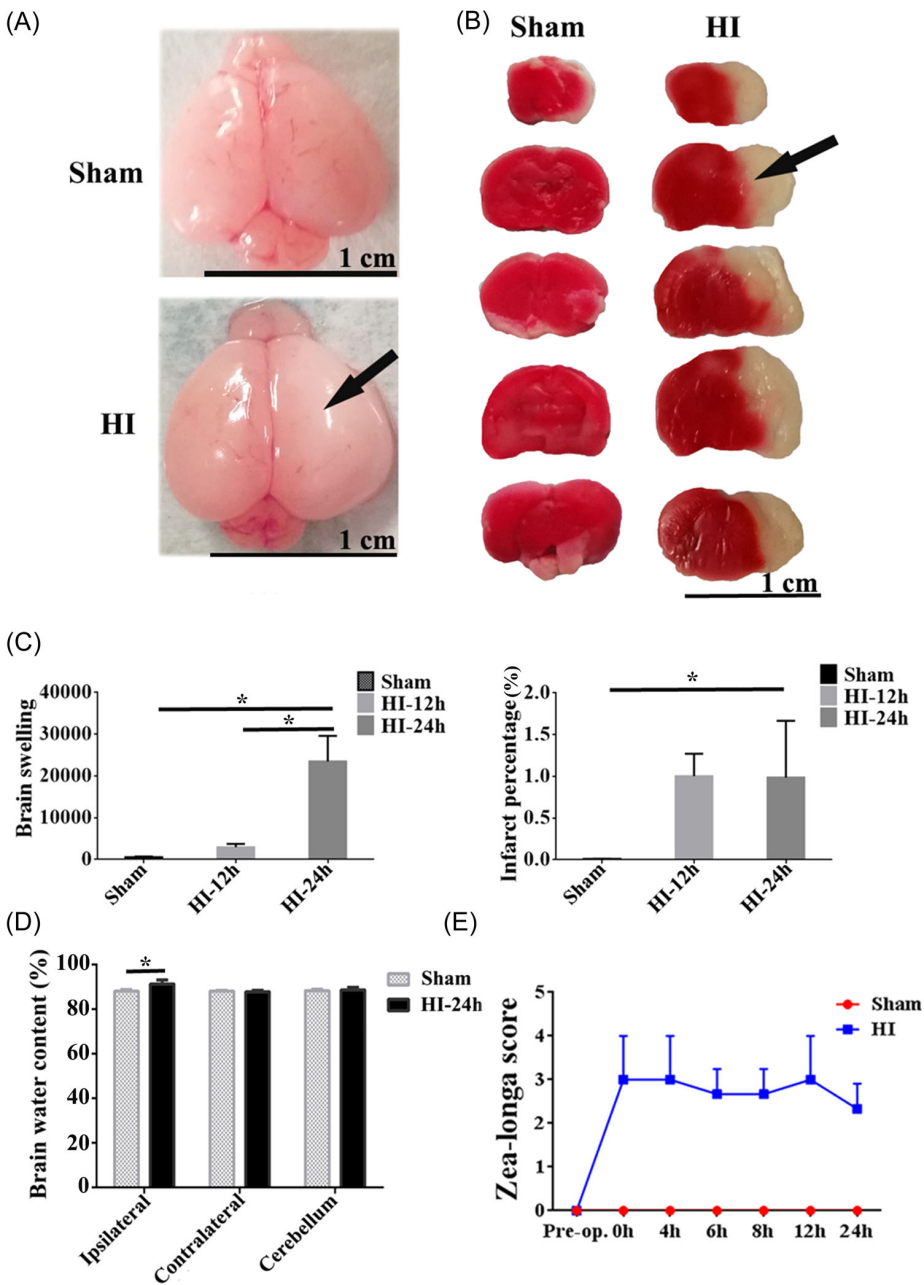
Successful establishment of the hypoxic‐ischemic (HI) model. (A) Representative images of the brain morphology in the sham and HI group. Scale bar = 1 cm. The black arrow indicates the swelling area of the ipsilateral brain. (B) Triphenyl tetrazolium chloride (TTC) staining images indicate the cerebral infarction of rats in the sham and HI groups. Scale bar = 1 cm. Black arrow indicates the infarcted area of the ipsilateral brain. (C) Quantification of the histograms for brain swelling and infarct percentage for sham, HI‐12 h, and HI‐24 h. (D) Brain water content for the ipsilateral hemisphere, contralateral hemisphere, and cerebellum in the Sham and HI groups. (E) Zea‐longa score was performed at 0, 4, 6, 8, 12, and 24 h on rats in sham and HI groups. HI‐12 h: 12 h after hypoxia‐ischemia; HI‐24 h: 24 h after hypoxia‐ischemia. All data are presented as mean ± SD, *n* = 6/group. *p < 0.05. [Color figure can be viewed at wileyonlinelibrary.com]

Brain injury (including swelling of the brain, water content of the brain, and cerebral infarction) was detected after the operation (20% mortality rate). The swelling of the brain and clear cerebral infarction were shown on the ipsilateral hemisphere of the HI group compared to the sham (Figure 1A,B). It was found that brain swelling markedly increased post‐HI, and there was a significant difference in the HI‐24 h group compared with that of the sham group. In addition, brain swelling at 24 h post‐HI was severer compared to 12 h post‐HI (*p* < 0.05, Figure 1C). The area of cerebral infarction was significantly increased at 12 and 24 h post‐HI as indicated by the infarct percentage (*p* < 0.05, Figure 1C). The water content of the brain was quantified, and it was markedly increased on the ipsilateral side in the HI group compared to the sham group (*p* < 0.05, Figure 1D). However, there was no obvious difference in the HI‐contralateral and HI‐cerebellum groups (Figure 1D). The Zea‐longa scores of HI rats were elevated at 24 h postinjury relative to those in the sham group (Figure 1E).

### 3.2 Morphological changes of brain tissues after HI

**Figure 2:**
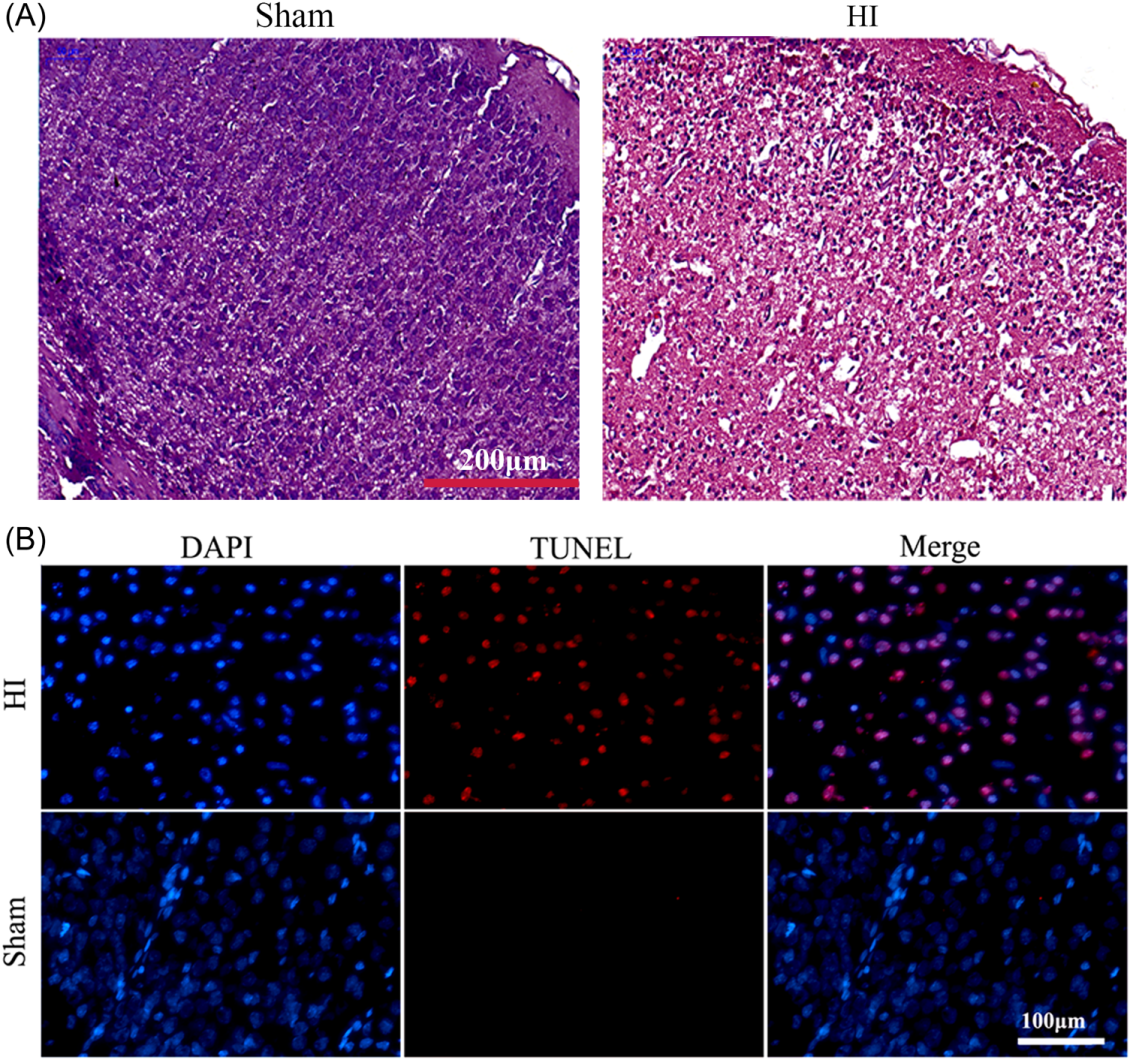
Morphological changes and apoptosis in the brain following the induction of HI in neonatal rats. (A) Representative H&E staining images showing morphological alteration in the sham and HI group. Scale bar = 200 μm. (B) Representative TUNEL staining images of the brain in the sham and HI group. Blue fluorescence indicates the location of the nucleus, and red fluorescence represents apoptotic cells. Scale bar = 100 μm. HI, hypoxia and ischemia; H&E, hematoxylin and eosin; DAPI: 4′, 6‐diamidino‐2‐phenylindole; TUNEL, terminal‐deoxynucleoitidyl transferase‐mediated nick end labeling. *N* = 6/group. [Color figure can be viewed at wileyonlinelibrary.com]

H&E staining was employed to assess the morphological changes of the brain tissues after HI insult. Obvious brain swelling was observed in the HI group, along with swelling or necrotic cells and illegible borders of cells in the HI group (Figure 2A). To explore the cause of neurological deficits in HI rats, we also detected apoptosis by TUNEL staining, and we found that there was a marked increase in the number of TUNEL‐positive cells in the HI group compared to the sham group (Figure 2B).

### 3.3 IL‐6 expression was increased in the cortex and hippocampus after HI

**Figure 3:**
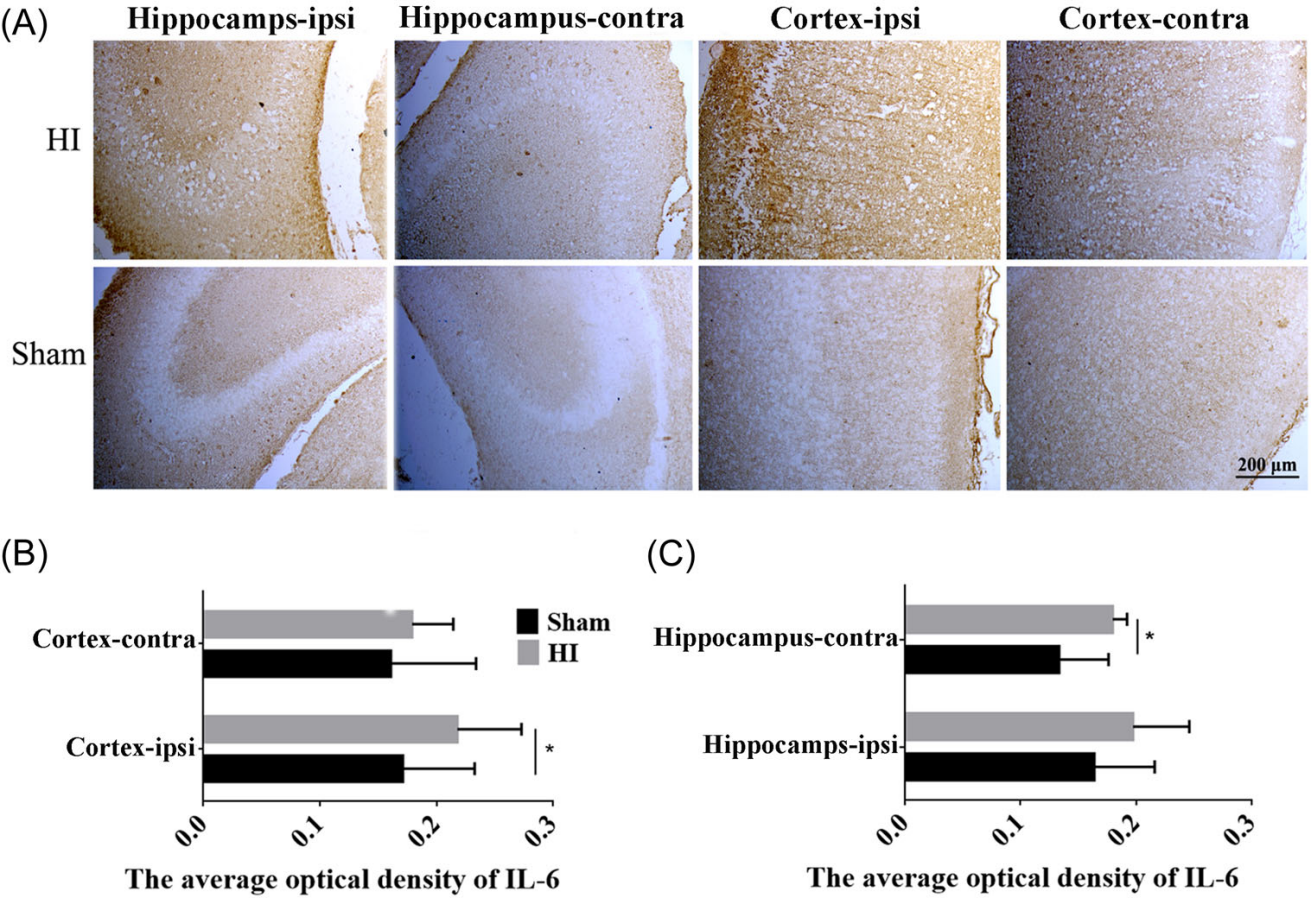
Immunohistochemical detection of interleukin (IL)‐6 positive cells in the hippocampus and cortex. (A) The location and the expression of IL‐6 in the hippocampus and cortex in the sham and HI groups detected via immunohistochemistry. Scale bar = 200 μm. (B, C) Quantitative analysis of IL‐6 levels in the right and left hippocampus and cortex between two groups. HI, hypoxia‐ischemia; ipsi, ipsilateral hemisphere; contra, contralateral hemisphere. All data are presented as mean ± SD, *n* = 6/group. *p < 0.05. [Color figure can be viewed at wileyonlinelibrary.com]

Immunohistochemistry was performed to semi‐quantify the level of IL‐6 in the hippocampus and cortex in the sham versus the HI rats. The results demonstrated that the number of IL‐6‐positive cells in the hippocampus and cortex increased significantly in the HI group compared to the sham group (Figure 3A,B, *p* < 0.05). In addition, the number of IL‐6 positive cells in HI rats was higher in the ipsilateral cortex and hippocampus compared to the respective contralateral sites (Figure 3B,C).

### 3.4 The expression of IL‐6 mRNA and protein was increased after HI in the ipsilateral cortex and hippocampus

**Figure 4:**
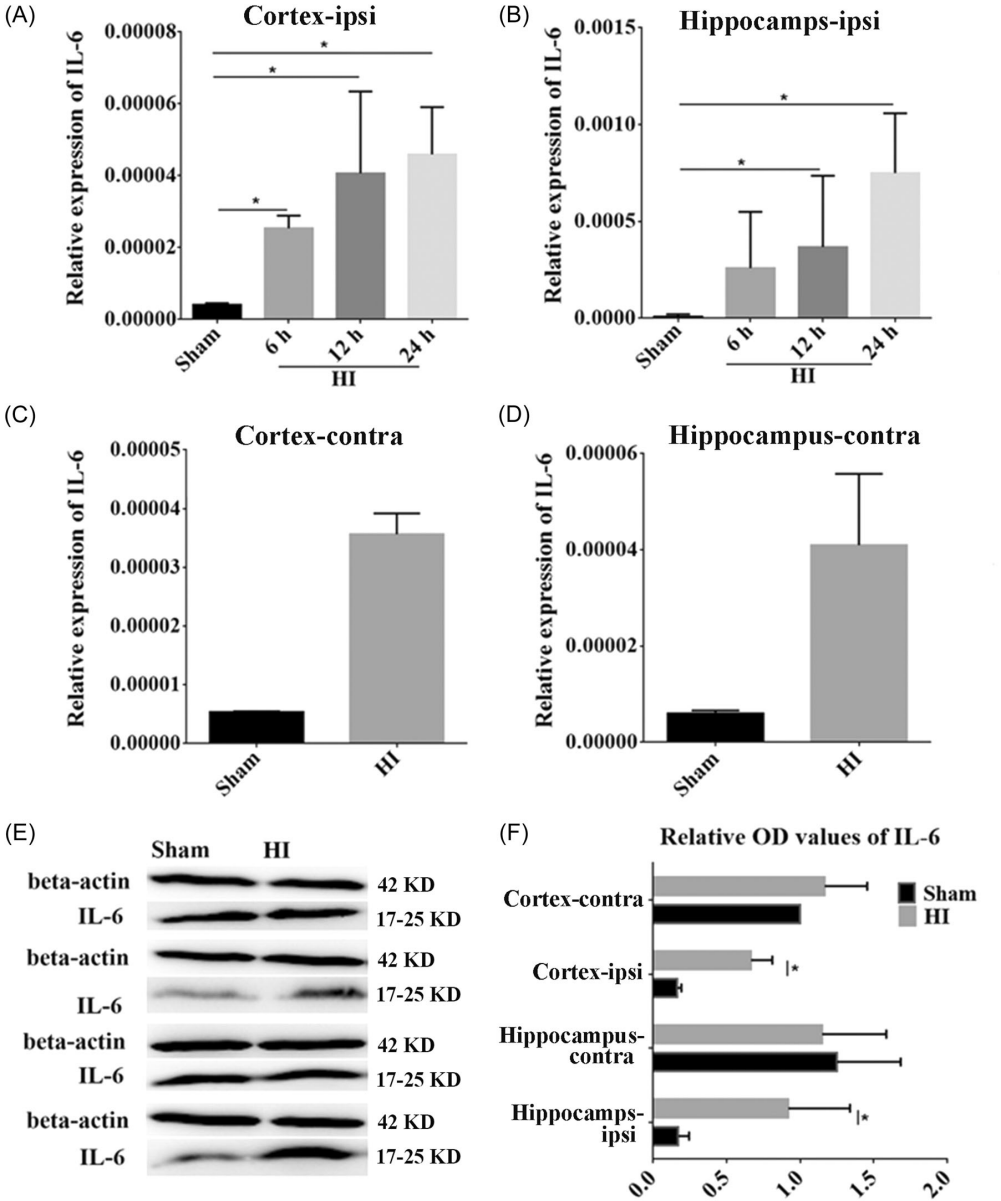
Alteration of interleukin (IL)‐6 expression in the hippocampus and cortex after HI. (A–D) The expression of IL‐6 mRNA in the cortex and hippocampus revealed by RT‐qPCR. (E, F) The protein expression of IL‐6 after HI in the cortex and hippocampus. HI, hypoxia‐ischemia; ipsi, ipsilateral hemisphere; contra, contralateral hemisphere. All data were presented as mean ± SD, *n* = 6/group. *p < 0.05.

The expression of IL‐6 mRNA in the ipsilateral hippocampus and cortex was examined by RT‐qPCR. We found that the levels of IL‐6 mRNA were increased significantly at 6, 12, and 24 h post HI in the ipsilateral cortex (*p* < 0.05, Figure 4A), and at 12 and 24 h post‐HI in the ipsilateral hippocampus (*p* < 0.05, Figure 4B) compared to the corresponding time in the sham group. In contrast, there was some change in IL‐6 mRNA expression in the contralateral cortex and hippocampus post HI compared to the sham group (Figure 4C,D), but without statistical significance. In addition, WB was performed to identify changes in IL‐6 protein expression. It was found that the expression of IL‐6 protein was increased after HI; thus congruent with mRNA expression (*p* < 0.05, Figure 4E,F).

### 3.5 The neurons and GFAP were in the vicinity of IL‐6

**Figure 5:**
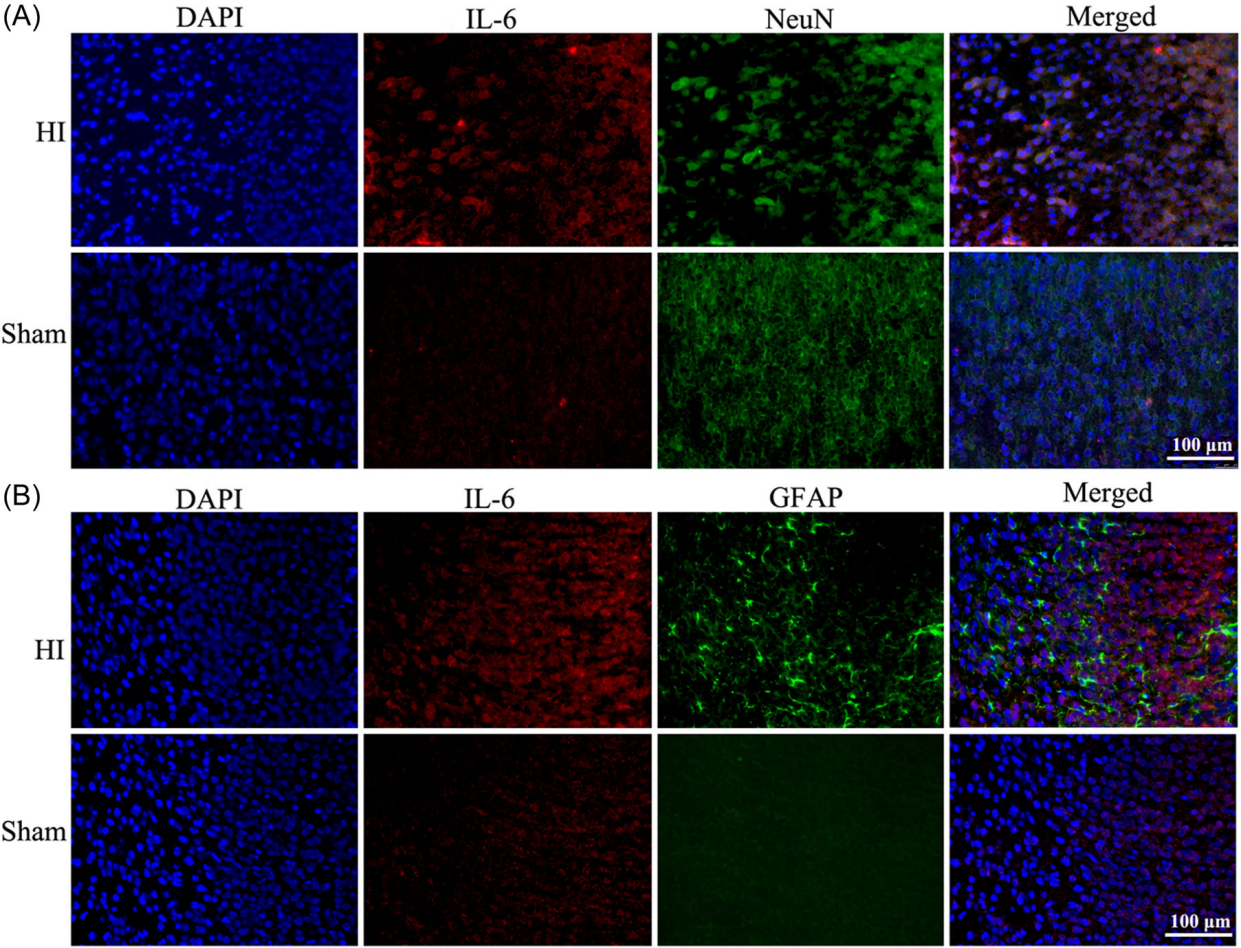
Location of interleukin (IL)‐6 in the neuron and astrocytes after HI. (A) Double immunofluorescence staining of IL‐6 and NeuN in the brain tissues from the sham and HI groups. Blue fluorescence indicates the location of the nucleus, red fluorescence represents IL‐6‐positive cells, and green fluorescence indicates NeuN‐positive cells (neurons marker). (B) Double Immunofluorescence staining of IL‐6 and GFAP in the brain tissues from HI and sham groups. The nuclei were stained with blue fluorescence, IL‐6 was stained with red fluorescence, and GFAP was stained with green fluorescence. DAPI: 4′,6‐diamidino‐2‐phenylindole; GFAP, glial fibrillary acidic protein; NeuN, hexaribonucleotide binding protein‐3. Scale bar = 100 μm, *n* = 6/group. [Color figure can be viewed at wileyonlinelibrary.com]

Immunofluorescence staining was performed to confirm the location of IL‐6 in the cortex. As shown, the increased IL‐6 expression after HI was localized to the neurons (Figure 5A). In addition, the expression of GFAP was elevated in the cortex of HI rats compared to the sham (Figure 5B).

### 3.6 Effective interference of the IL‐6 gene

**Figure 6:**
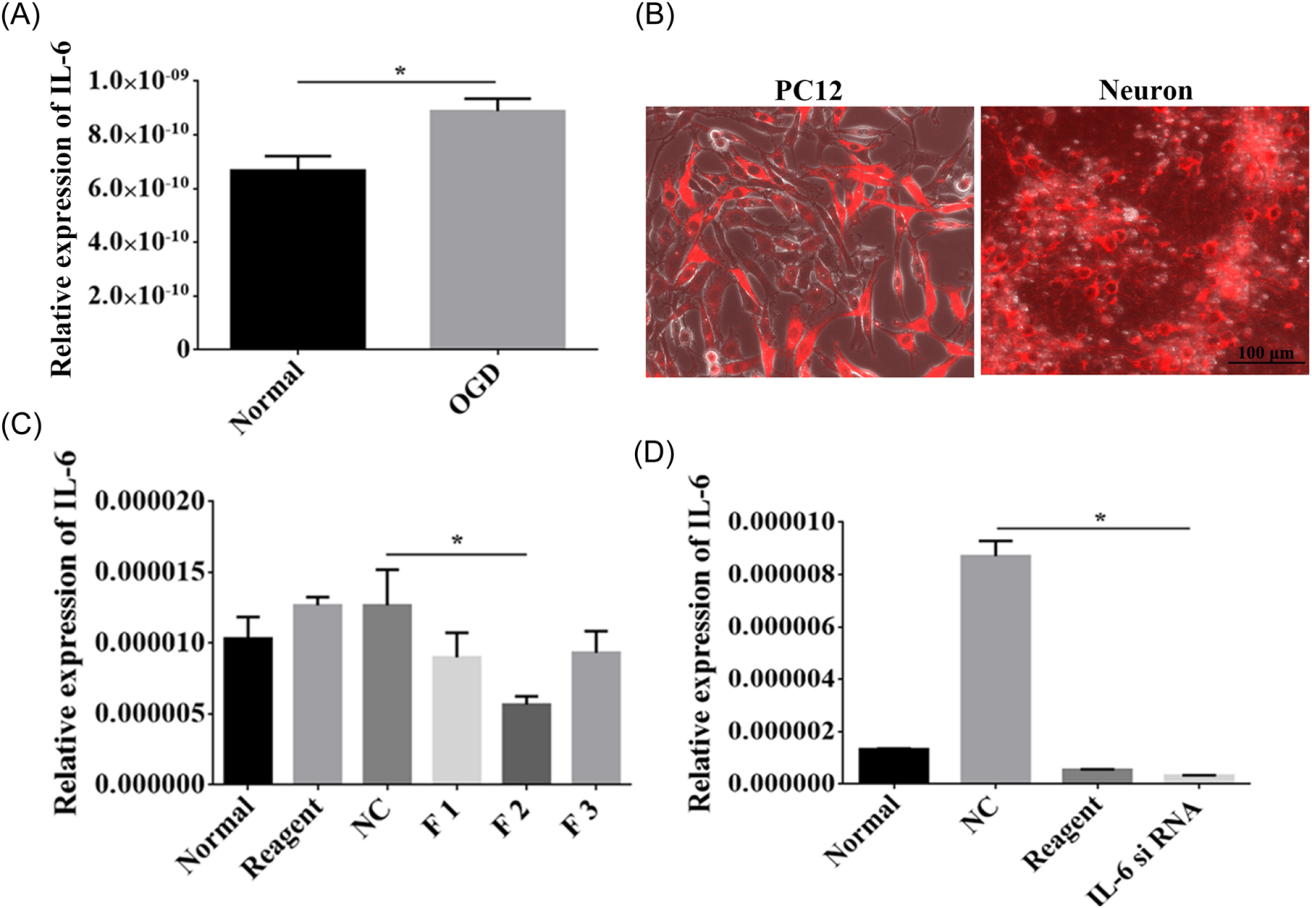
Testing the efficacy of interleukin (IL)‐6 mRNA interference fragments. (A) The expression of IL‐6 in cortical neurons under normal and OGD conditions. (B) PC12 cells were successfully transfected with F1, F2, and F3 interference fragments, and the F2 fragment was successfully transfected into cortical neurons. Scale bar = 100 μm. (C) Quantitative analysis of the interference efficiency of F1, F2, and F3 fragments. (D) RT‐qPCR verification of IL‐6 expression after siRNA transfection. Scale bar = 100 μm. OGD, oxygen‐glucose deprivation; siRNA, small interfering RNA. F1: treatment with No.1 siRNA fragment; F2: treatment with No.2 siRNA fragment; F3: treatment with No.3 siRNA fragment. All data are presented as mean ± SD, *n* = 6/group. *p < 0.05. [Color figure can be viewed at wileyonlinelibrary.com]

The expression of IL‐6 was upregulated after OGD treatment (*p* < 0.05, Figure 6A). To determine the effective interference fragment on the IL‐6 gene, we transfected F1, F2, and F3 interference fragments into PC12 cells respectively (Figure 6B). Subsequently, RT‐qPCR was utilized to detect the interference efficiency, and we found that F2 was the most efficient interference fragment (*p* < 0.05, Figure 6C). Furthermore, the F2 interference fragment was transfected into cortical neurons, which revealed that it could significantly inhibit IL‐6 gene expression (Figure 6B), which was further verified by RT‐qPCR (*p* < 0.05, Figure 6D).

### 3.7 The growth of cortical neurons was promoted after interference with IL‐6 under OGD conditions

**Figure 7:**
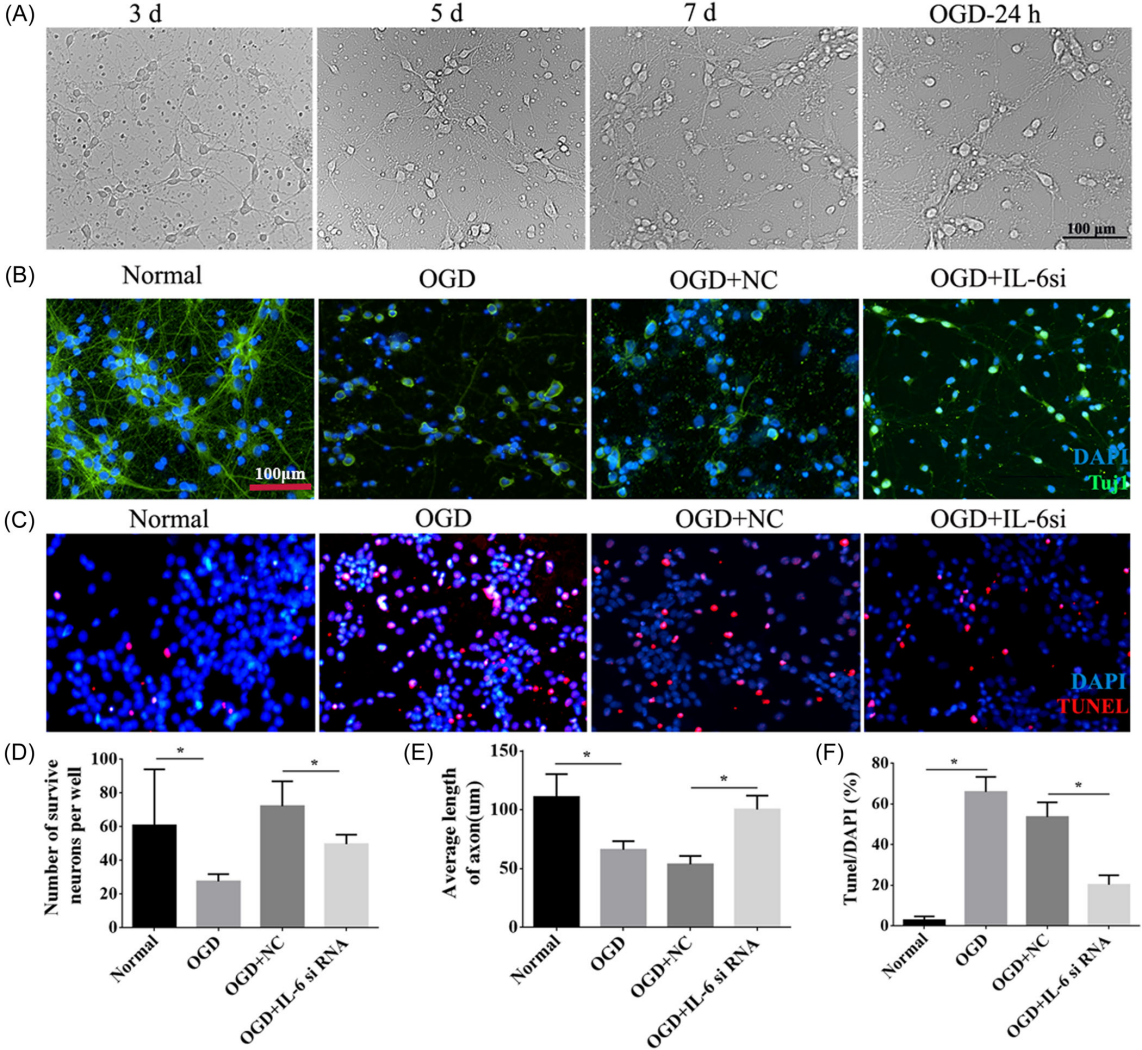
Effects of interleukin (IL)‐6 interference on cerebral OGD cortical neurons. (A) Bright‐field pictures (200x) of normal neurons 3, 5, 7 days, and OGD neurons at 24 h. (B, C) The growth and apoptosis of neurons were observed by immunofluorescence staining of Tuj1 and TUNEL among normal, OGD, OGD + NC, and OGD + IL‐6si groups. Scale bar = 100 μm. DAPI: nucleus marker (blue); Tuj1: neuron marker (green); TUNEL: apoptosis marker (red). (D–F) Statistical analysis showed the number of neurons, the average length of axons, and apoptosis of neurons after interference with IL‐6 under OGD conditions in these groups. NC, negative control; OGD, oxygen‐glucose deprivation; Tuj1, neuronal class III β‐tubulin; TUNEL, terminal‐deoxynucleoitidyl transferase mediated nick end labeling. IL‐6 si: IL‐6 silencing. All data are presented as mean ± SD, *n* = 6/group. *p < 0.05. [Color figure can be viewed at wileyonlinelibrary.com]

Normal cells were homogenously distributed within or along the walls, of which some emerged as spherical buds, some as laminar aggregates, and others dispersed as single cells over time (Figure 7A). While after OGD, neurons were damaged and distributed disorderly. Double immunofluorescence staining for Tuj1 and TUNEL was conducted to investigate the role of IL‐6 in neuronal growth and survival. The outcomes revealed that the number of neurons and the length of axons were significantly reduced after OGD (*p* < 0.05, Figure 7B,D,E), and apoptotic cells were in a larger number in the OGD group compared to the normal group (*p* < 0.05, Figure 7C,F). However, IL‐6 downregulation increased the neurons' numbers and axonal length and decreased the number of apoptotic cells in comparison to the OGD + NC group (*p* < 0.05, Figure 7B–F).

### 3.8 BAX and caspase 3 interacted with IL‐6

**Figure 8:**
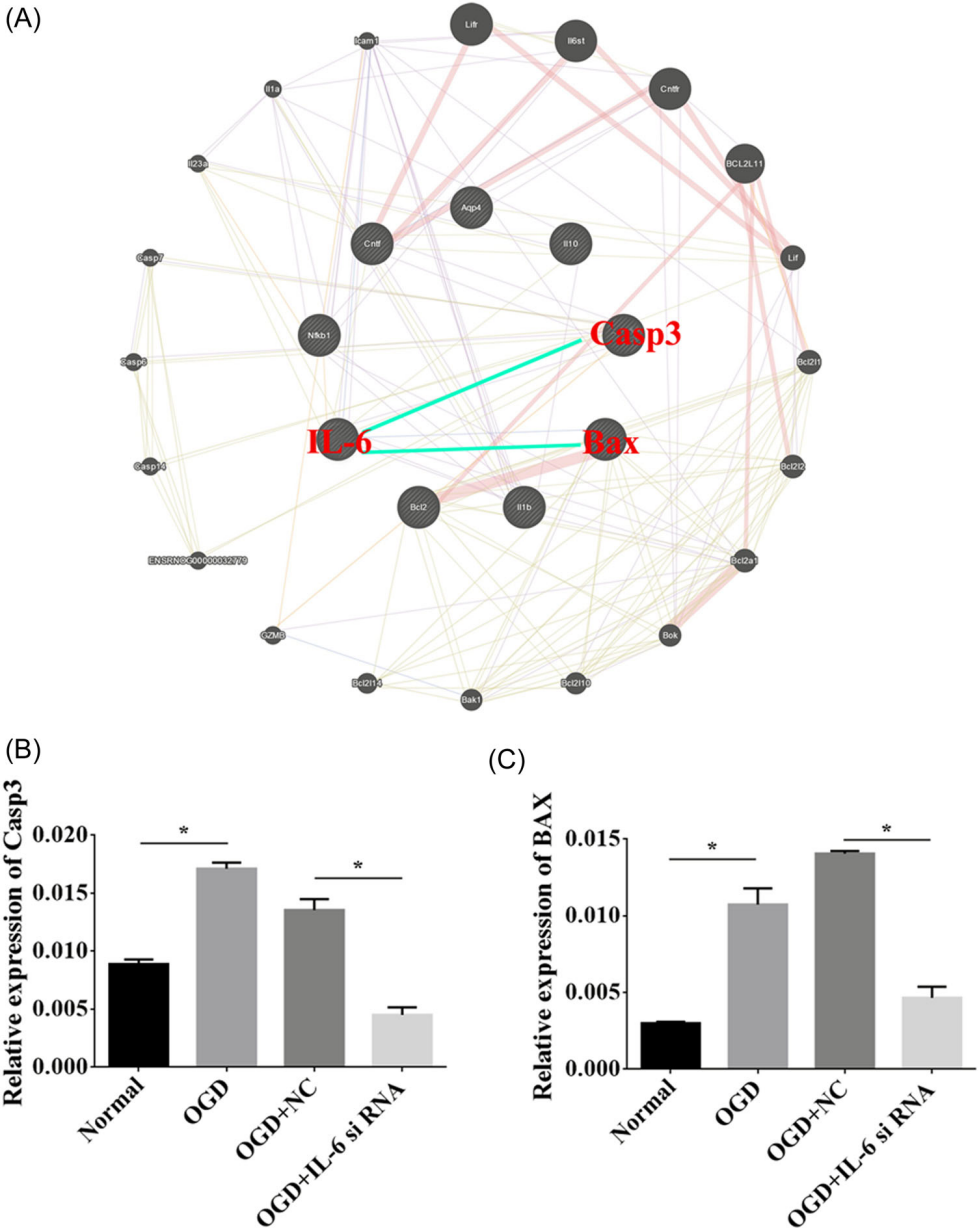
The interaction of interleukin (IL)‐6 with BAX and caspase 3. (A) The protein‐protein interaction network analysis of IL‐6, BAX, and caspase 3. (B, C) Relative expression of BAX and caspase 3 in OGD, OGD + NC, OGD + IL‐6 siRNA groups. BAX, Bcl‐2‐associated X protein; IL‐6si, IL‐6 silencing; OGD, oxygen‐glucose deprivation; NC: negative control. All data were presented as mean ± SD, *n* = 6/group. *p < 0.05. [Color figure can be viewed at wileyonlinelibrary.com]

An interaction network was generated for the relationship of IL‐6 with BAX and caspase 3 using GeneMANIA software (Figure 8A). We performed RT‐qPCR to elucidate the effects of IL‐6 downregulation on the expression of BAX and caspase 3, and we found that the levels of BAX and caspase 3 were prominently elevated in the OGD group. However, BAX and caspase 3 were depressed in the OGD group that received IL‐6 siRNA compared to the negative control (*p* < 0.05, Figure 8B,C).

### 3.9 The relative expressions of BAX and caspase 3 were associated with IL‐6 siRNA

**Figure 9:**
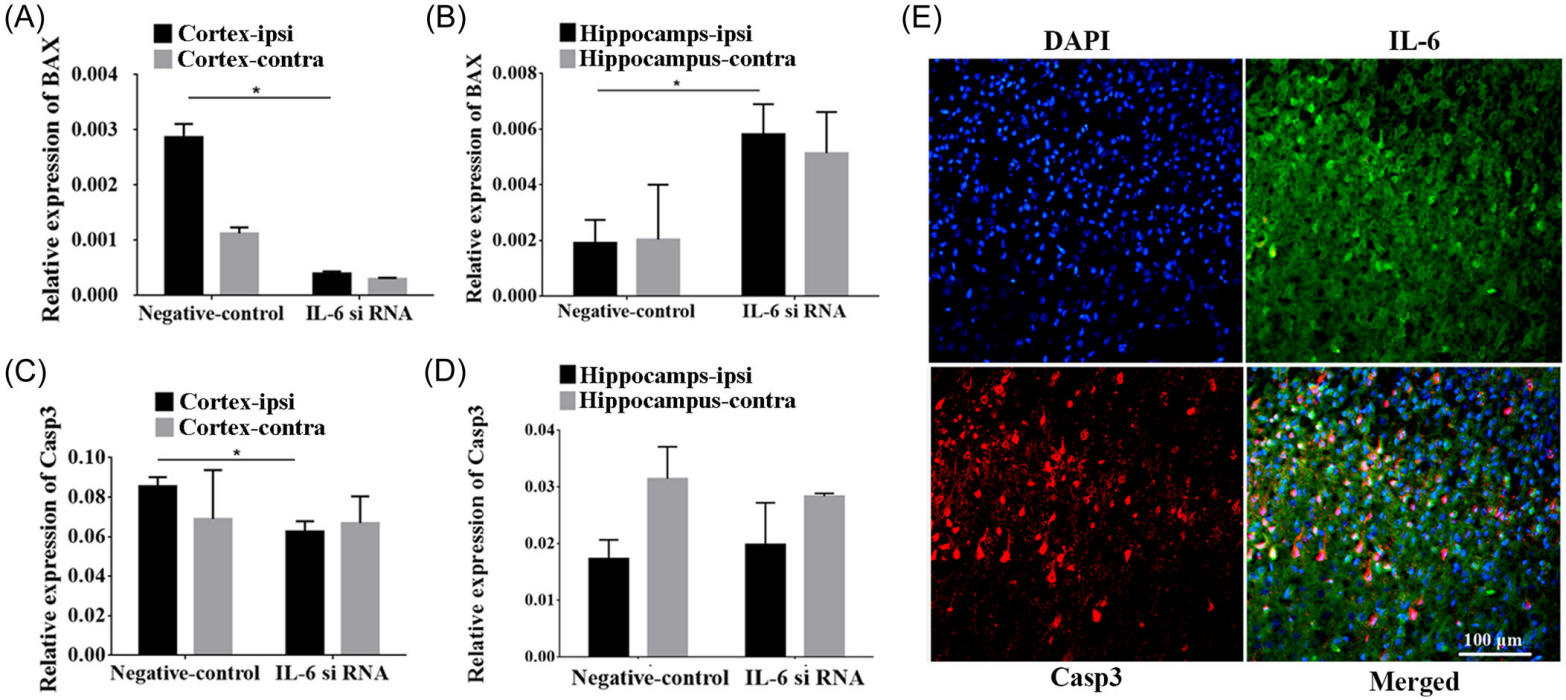
The impact of interleukin (IL)‐6 silencing on the expression of BAX and caspase 3. (A–D) The level of BAX and caspase 3 expression in the cortex and hippocampus after IL‐6 siRNA treatment. (E) Double immunofluorescence labeling demonstrated colocalization for IL‐6 (green) and caspase 3 (red). Scale bar = 100 μm. BAX, Bcl‐2‐associated X protein; Casp3, caspase 3; DAPI, 4′,6‐diamidino‐2‐phenylindole; ipsi, ipsilateral hemisphere; contra, contralateral hemisphere. All data were presented as mean± SD, *n* = 6/group. *p < 0.05. [Color figure can be viewed at wileyonlinelibrary.com]

Functional experiments exhibited that BAX expression was prominently decreased in the ipsilateral cortex but increased in the ipsilateral hippocampus after IL‐6 silencing in comparison to the negative control group (*p* < 0.05, Figure 9A,B). No significant difference in BAX after IL‐6 silencing was revealed both in the contralateral cortex and hippocampus. In addition, the relative expression of caspase 3 in the ipsilateral cortex was significantly reduced after the knockdown of IL‐6 compared to that of the negative control (*p* < 0.05, Figure 9C), however, there was no statistical difference in caspase 3 in the contralateral cortex as well as hippocampus of two hemispheres (Figure 9C,D). Double immunofluorescence labeling demonstrated the co‐localization of IL‐6 (green) with caspase 3 (red) (Figure 9).

## 4 DISCUSSION

This study demonstrated that HI‐induced neurological deficits, brain swelling, and infarction were accompanied by upregulation of IL‐6 expression in the ipsilateral cortex and hippocampus. IL‐6 was localized in the neurons, and its downregulation inhibited cellular apoptosis and enhanced neuronal outgrowth after OGD. In addition, IL‐6 interacts with BAX and Caspase 3 based on an interaction network generated by GeneMANIA software, and IL‐6 silencing depressed the levels of BAX and caspase 3 in OGD neurons, and in the cortex and hippocampus of neonatal rats. Altogether, our findings suggest that caspase 3 and BAX might be regulated in IL‐6‐induced pathology, and thus they could be potential therapeutic targets for the treatment of NHIE.

### 4.1 HI induced brain injury and neuronal damage

In this study, we used cultured primary cortical neurons and postnatal rats (7‐day‐old) to establish HI models in vitro and in vivo. We examined the morphological variation of brain tissues and neurons following HI, and brain infarction, cellular swelling, inflammatory cell infiltration, and cellular necrosis were observed in the HI group. Moreover, the degree of neurological dysfunction was present in a time‐dependent manner under HI intervention. These results suggested brain injury and deterioration of neurological function in rats after HI, consistent with what's reported in previous publications denoting that neonatal HI not only induces periventricular white matter injury known as periventricular leukomalacia and significant neuronal death,^24^, ^25^ but also stimulates cerebral microvascular responses and BBB damage,^26^, ^27^ ultimately leading to neurological functional deficits.^28^

### 4.2 Deceased level of IL‐6 involved in the neuroprotection against NHIE

In this study, we found that the mRNA expression level of IL‐6 was markedly increased following HI in the hippocampus and cortex, along with the augmentation of IL‐6 positive neurons in the hippocampus and cortex of HI rats. It has been reported that IL‐6 levels in the serum increased within the first 24 h after HI.^29^ Similarly, we found in this study the level of IL‐6 in the ipsilateral cortex exhibited elevating trend at 6, 12, and 24 h after HI injury. To explore the role of IL‐6 in NHIE, IL‐6 expression was interfered in vitro and in vivo. Immunofluorescent staining results demonstrated neuronal outgrowth was significantly enhanced and cellular apoptosis was prominently inhibited after IL‐6 silencing, indicating that downregulated IL‐6 plays a crucial and active role in the restoration of neuronal survival and growth in HI conditions. Recent studies have also found that hypoxic cortical neurons upregulated IL‐6 mRNA and protein expression.^30^ Moreover, some researchers suggested that breviscapine is effective in promoting neurological behavior after traumatic brain injury by suppressing levels of IL‐6.^31^ Thus it can be concluded from this study that IL‐6 may play its neuroprotective role in NHIE. However, it's contrary to the results of our previous study^32^ in which IL‐1β downregulation improved HI brain injury associated with IL‐6 upregulation. It has been suggested that IL‐6 might be released as a protective response after HI brain injury and is involved in the repair process in the sub‐acute stage of HIE.^33^ The difference between these studies might be attributed to the usage of the model at distinct HIE stages and different detection time points, which deserves more in‐depth comparative investigation in the future.

### 4.3 Neuroprotective effects of IL‐6 silencing in NHIE correlated with suppression of BAX and caspase 3

GeneMANIA network revealed that IL‐6 interacts with BAX and caspase 3. Simultaneously, qRT‐PCR verified that the levels of both caspase 3 and BAX were prominently decreased after IL‐6 siRNA treatment in OGD neurons. In addition, levels of caspase 3 and BAX in the cortex and hippocampus were significantly reduced after IL‐6 expression downregulation, implying that the presence of IL‐6 could naturally activate the expression of caspase 3 and BAX in brain development. In a separate study, Diazepam attenuated myocardial injury by inhibiting inflammatory release (CCR2, TNF‐α, and ILs), oxido‐nitrosative stress, and apoptosis (BAX and caspase 3), thus improving myocardial function.^14^ As known, HI brain injury is often delayed and involves both apoptotic and immune regulatory mechanisms.^34^ Some studies have shown that HI induces IL‐10 secretion from astrocytes to exert a paracrine‐induced antiapoptotic effect on injured neurons via the TLR2/NFκB signaling pathway, which may improve learning and memory dysfunction after ischemic injury.^35^ Regulating the levels of IL‐6, Fas, and BDNF in the brain to maintain reasonable levels was beneficial to neurological functional recovery.^36^ Previous researchers found that the neuroprotective effect of MSC transplantation in neonatal HI rats is partly attributed to IL‐6‐mediated antiapoptosis of injured astrocytes via the IL‐6/STAT3 signaling pathway.^37^ BAX is a proapoptotic protein responsible for mitochondrial apoptosis via the release of cytochrome C and formation of its apoptosome complex with apoptotic protease‐activating factor‐1.^38^, ^39^ Further, this apoptosome causes DNA fragmentation through activation of caspase 3 and promoting activity of caspase 3‐activated DNase enzyme.^40^ The findings of the present study also suggested that HI‐induced apoptosis in neurons is reflected by augmented BAX and caspase 3 protein expression in the cortex with the expression of IL‐6. Interestingly, silencing IL‐6 expression attenuated HI‐induced apoptosis in the cortex via suppression of BAX and caspase 3, depicting its antiapoptotic property.

## 5 CONCLUSIONS

Altogether, these findings suggested that IL‐6 may be a therapeutic target of NHIE. Our results unveiled a novel insight into developing genetic intervention strategies for the treatment of NHIE.

## AUTHOR CONTRIBUTIONS

*Conceptualization and supervision*: Lan‐Chun Zhang. *Methodology*: Xiu Yang and Isaac B. Deng. *Formal analysis and investigation*: Ke‐Han Liao. *Writing‐original draft*: Xiu Yang. *Writing review and editing*: Isaac B. Deng and Lan‐Chun Zhang.

## CONFLICT OF INTEREST

The authors declare no conflict of interest.

## ETHICS STATEMENT

All the experiments were carried out in accordance with the care and use of laboratory animals promulgated by the Ministry of Science and Technology of the People's Republic of China and approved by the Animal Care and Use Committee of Kunming Medical University (kmmu2019039) and were in compliance with the National Institutes of Health Guide for the Care and Use of Laboratory Animals.

## TRANSPARENCY STATEMENT

All the authors affirm that this manuscript is an honest, accurate, and transparent account of the study being reported; that no important aspects of the study have been omitted; and that any discrepancies from the study as planned (and, if relevant, registered) have been explained.

## Data Availability

Data of our study are available on reasonable request.
